# Personalized Image Classification by Semantic Embedding and Active Learning †

**DOI:** 10.3390/e22111314

**Published:** 2020-11-18

**Authors:** Mofei Song

**Affiliations:** School of Computer Science and Engineering, Southeast University, Nanjing 211189, China; songmf@seu.edu.cn

**Keywords:** image classification, semantic embedding, active learning

## Abstract

Currently, deep learning has shown state-of-the-art performance in image classification with pre-defined taxonomy. However, in a more real-world scenario, different users usually have different classification intents given an image collection. To satisfactorily personalize the requirement, we propose an interactive image classification system with an offline representation learning stage and an online classification stage. During the offline stage, we learn a deep model to extract the feature with higher flexibility and scalability for different users’ preferences. Instead of training the model only with the inter-class discrimination, we also encode the similarity between the semantic-embedding vectors of the category labels into the model. This makes the extracted feature adapt to multiple taxonomies with different granularities. During the online session, an annotation task iteratively alternates with a high-throughput verification task. When performing the verification task, the users are only required to indicate the incorrect prediction without giving the exact category label. For each iteration, our system chooses the images to be annotated or verified based on interactive efficiency optimization. To provide a high interactive rate, a unified active learning algorithm is used to search the optimal annotation and verification set by minimizing the expected time cost. After interactive annotation and verification, the new classified images are used to train a customized classifier online, which reflects the user-adaptive intent of categorization. The learned classifier is then used for subsequent annotation and verification tasks. Experimental results under several public image datasets show that our method outperforms existing methods.

## 1. Introduction

Image classification is a fundamental and important task in computer vision and artificial intelligence. With the explosive growth of labeled image data, deep convolutional neural networks (DCNN) have become the mainstreaming method and achieved impressive performance on closed datasets [1,2,3,4], where the taxonomy of the datasets is pre-defined. However, in a practical image classification task, it is usually impossible to fix the taxonomy in advance, since different users have their own requirement. Presently, people can store a lot more images on their own personal computers and smartphone, which makes it crucial to design a personalized classification system. Due to the diversity of users’ preferences, personalized image classification is very challenging. It is impractical to extract a flexible feature representation for all the taxonomies. Moreover, the lack of specific supervision makes it difficult to pre-train a customized classifier.

To address this issue, some methods [5,6] first extract many features from different views to cover diverse requirements, and then use online user intervention to confirm and refine the class label of every image interactively. As a result, the users can control the classification of the given image collection in a user-adaptive way. The key to these interactive methods is to reduce user effort when classifying a large collection. By treating the image set as streaming data, these methods train a customized classification model by online learning. Thus, the performance of the learned model increases progressively along the iterative process, which reduces the user’s burden for subsequent categorization significantly. However, there are two main problems in these methods.

The first problem is the adaptability of the image representation. especially, these methods [5,6] directly concatenate the appearance and semantic features into a high-dimensional vector representation, which is very inefficient. Moreover, the semantic feature only covers the discrimination information of one certain taxonomy. Thus, it cannot satisfy the diverse requirement of the personalized classification task. The second problem is about the cold-start issue of the classifier. Learning a reliable classifier requires enough training data. During the early stage of the iterative process, the size of training images is too limited to satisfy the requirement of classifier learning. This leads to many unsatisfactory automatic predictions, which increases a heavy effort from the user for label refinement.

To solve these two problems, our idea is to introduce semantic embedding [7] and active learning [8] into the personalized image classification framework. Our motivation for introducing semantic embedding is based on the following observation: the differences between the taxonomies of multiple users usually focus on the granularities of the category concept, and most taxonomies follow the hierarchical structure of the semantic concept. For example, some users might group the two categories ‘dog’ and ‘cat’ into the category ‘animal’, but nobody would combine the categories ‘dog’ and ‘airplane’ into one group. Thus, the feature is required to reflect the similarity degree between different category labels, which is implicit in the hierarchical structure of the semantic concept. To extract such features, we use semantic embedding to encode the similarity between the category labels for representation learning. The performance of the classifier can then be improved when facing multiple taxonomies with different granularities. The goal of introducing active learning is to alleviate the cold-start situation. especially, we use active learning to sample the most representative images and ask the users to determine their categories first. By training these informative images as early as possible, the performance of the classifier improves faster, which saves user effort by avoiding a large number of manual refinement operations.

Accordingly, this paper presents an interactive image classification system by incorporating semantic embedding and active learning for personalized applications. Our system includes two sessions: offline feature learning and online image classification. The offline session trains a specific DCNN model with semantic embedding to generate a flexible feature representation. Based on this representation, the online session takes an image collection as input. The given collection can then be classified by incorporating active learning, automatic classification, and user interaction as an iterative manner.

For each image of the given collection, we extract its feature representation by a pre-trained deep model, which is learned by incorporating the semantic-embedding method. The semantic-embedding method can measure the similarity between the words by turning them into vector representations. In our model training step, we use a pre-trained semantic-embedding model to transform every discrete category label to a continuous vector. The goal of the label transformation is to enhance the labels’ information for similarity measurement, which is not applicable by the discrete ones. By using both the discrete and continuous labels, the deep model is then obtained by minimizing the combination of the cross-entropy loss and the log-ratio loss [9]. The cross-entropy loss is to measure the common classification loss, while the log-ratio loss is to penalize the discrepancy between the ratio of continuous label distances and that of the learned feature distances. Based on our loss function, we can encode useful similarity relations between different semantic categories into the learned deep model. The online interactive classification then starts after the feature of each image is extracted by this deep model.

During the online session, our system first selects some images from the collection for interactive classification, since it is not convenient to handle a large number of images manually at once. Then, a classification model is employed to generate the classification of the selected images automatically, which are shown to the user for interactive manipulation. Our system provides two interactive interfaces: annotation and verification. The user labels the images one by one during the annotation, while confirms the automatic prediction of the classifier group by group during the verification.

After user intervention, the new classified images are used as the incremental training data for classifier learning by the soft confidence weighted learning method [10]. The image selection, classification generation, user intervention, and classifier training are repeated until all the images are classified. The main advantage of our system is that we propose an active learning algorithm to select which images to be annotated or verified. Our motivation is that the selecting result has a major impact on interactive efficiency, as these selected images determine the performance of the classifier. To realize the image selection, our active learning algorithm formulates it as a combinatorial optimization problem. The optimization variables are the candidate annotation or verification set, while the objective function is the interactive classification efficiency. Intuitively, the classification efficiency is defined as the number of classified images per second generated by our system. To optimize the efficiency objective, a beam-searching algorithm is used to obtain the optimal annotation or verification set.

**Contribution.** The contribution of this work contains three aspects: first, we present a novel interactive system by integrating semantic embedding, active learning, online learning into a unique framework. Second, we propose a feature learning algorithm for personalized image classification by using semantic embedding, which is realized by a novel loss function as the combination of the cross-entropy loss and the log-ratio loss. Third, we design an active learning algorithm that dynamically selects images for manual annotation and verification, which saves user effort significantly in an online multi-class classification scenario.

The work is an extension of our preliminary research [11]. The extensions are summarized as follows: first, we introduce the idea of semantic embedding for feature learning, which improves the flexibility of image representation. Second, to produce a more reliable prediction, we extend the classification framework by blending of a common classifier and a personalized one. Third, we conduct an exhaustive experiment and find that the extended work improves the efficiency by 20.0% compared with the conference version.

## 2. Related Work

There is a significant body of research on image classification. In this section, we discuss related work on image classification, personalized classification, and active learning.

### 2.1. Image Classification

Image classification has attracted a lot of researchers in the area of computer vision and artificial intelligence. The traditional methods first extract the handcrafted features from images by various descriptors [12,13], and then train a shallow classifier. The accuracy of these methods profoundly depends on the design of the feature, but these handcrafted features are usually difficult to generalize well to varied image collections. With the recent rapid development of deep learning, DCNN shows state-of-the-art performance on image classification [14]. The main idea is to replace the handcrafted image features employed in the traditional methods with data-driven learned ones [1,2,3,4]. However, these networks are usually trained to target a fixed taxonomy, which is difficult to satisfy the requirement of the personalized image classification task. In this paper, we also use DCNN to learn the image representation, but introduce semantic embedding to improve the adaptability of the representation for personalized classification.

### 2.2. Personalized Classification

While most existing image classification methods classify the image collection with a common taxonomy, the result of personalized classification depends on the preference of the user. Currently, a lot of images can be collected by individual users, which makes it essential to develop effective personalized image classification methods. According to whether user intervention is required, existing work can be divided into automatic methods and interactive methods. The automatic methods [15,16] learn the personalized classifier based on personal collections or tagging history in an offline manner. However, a large amount of data are required to be annotated according to a specific user’s preference in advance. Another way [17,18] is to collect personalized information progressively during user interaction. However, most existing methods are not very efficient, since users are asked to label the examples one by one. The most related works are interactive image classification method [5,6]. Instead of providing one image once, these methods provide a group of images and their category prediction at one time, which allows the user to interactively classify a collection of images in a more high-throughput way. However, the limited training data of the early stage cannot lead to a reliable classifier, which increases the user burden significantly. In contrast, we design an active learning algorithm to improve the performance of the classifier quickly, which saves user effort as much as possible.

### 2.3. Active Learning

Active learning is a machine learning technique that searches the most representative training samples to reduce the label cost [8]. This is very useful when labels are scarce, and has shown to require fewer training data in image categorization [19,20] and soldering defect detection [21]. For example, Wang et al. [19] propose a cost-effective sample selection method to choose many high-confidence samples for deep learning. The goal of these methods is to increase the performance of the classifier with the fewest labeled images. Our goal is different, and we focus on classifying every image under manual confirmation in the shortest time. Recently, active learning is employed to 3D shape classification or annotation. Yi et al. [22] propose an active framework for annotating massive 3D shape datasets. Song et al. [23,24] further realize iterative 3D shape classification by combining active learning and online learning. Our method is inspired by these work, and extending them to support personalized image classification. As the image contains more information than 3D shape, we propose several strategies to make it work on the image domain. First, we propose an image feature learning method to create an effective image descriptor for personalized classification. Second, the dimension of the image feature is much higher than that of the 3D shape. In the area of 3D shape classification, the state-of-the-art MVCNN [25] only requires 128-dimensional vector to obtain the classification accuracy more than 90% in ModelNet40 [26]. With its low dimension and high discrimination, this yields very good performance for interactive 3D shape classification [23,24]. However, for image classification, as the variation between images is much larger [27], the dimension of the feature layer is above 2000 in the typical network model. To reduce the influence caused by the curse of-dimensionality, we design a classification prediction algorithm based on a conditional random field (CRF).

## 3. Overview

**Figure 1:**
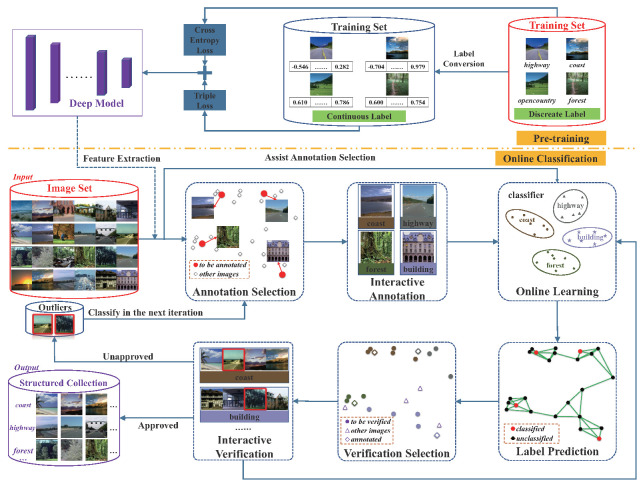
The pipeline of our framework. The red and purple boxes show the input and output of the two sessions.

The input to our system is an image set without any label. Our system generates several disjoint groups of images with light supervision in a user-adaptive manner, where the label of every image is explicitly determined by the user. Figure 1 illustrates the overall pipeline of our framework.

There are two sessions in our framework: the pre-training session and the online classification session. The goal of the pre-training session is to learn a deep model for feature extraction. The input of the pre-training session is a set of training images, which are pre-labeled in a common taxonomy. For each category label, we extract a semantic-embedding vector using the pre-trained GloVe model [7]. This transforms the pre-defined discrete label into the continuous label, which can describe the similarity degree between different categories. Based on the discrete and continuous labels, we propose a novel loss function as the sum of the cross-entropy loss and the log-ratio loss to realize the model training. The achieved deep model is then used to extract the feature of every input image for online classification. Moreover, the deep model contains a SoftMax classifier layer, which is also used for online classification. When the pre-training session finishes, the deep model is not updated.

During the online classification session, our approach classifies the given collection group by group iteratively. Each iteration contains two user tasks: annotation and verification. The annotation asks the user to label the images that are important for classifier learning, while the verification asks the user to quickly confirm the prediction on a large number of images with high-confidence. There are four steps in each iteration: annotation/verification selection, interactive annotation/verification, online learning, and label prediction.

**Annotation/verification selection.** To maximize classification efficiency, our system introduces a dynamic image selection strategy for user interaction. To obtain the optimal images, we design a unified utility function as the optimization objective for both annotation and verification tasks. The objective function measures the classification efficiency, which depends on two terms: the number of the classified images and the required manual timing. The timing is measured as the probabilistic weighted sum of the statistics timing of each interactive operation, such as refining the label of a certain image, or confirming the prediction. Based on this measurement, we perform image selection by efficiency maximization as an optimal subset searching process.

**Figure 2:**
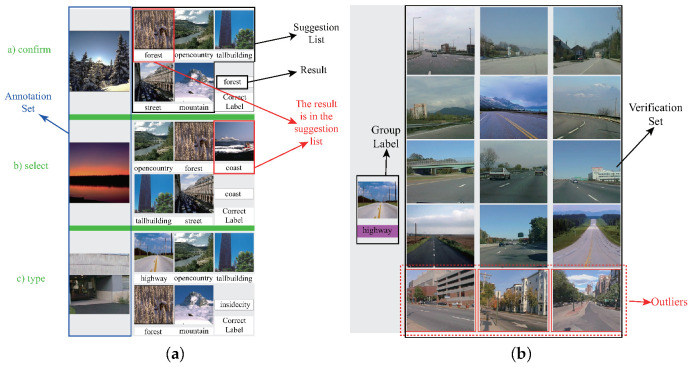
User task. (**a**): annotation task; (**b**): verification task.

**Interactive annotation/verification.** Our system contains two interfaces for the annotation and verification task. For the annotation task, our interface shows the selected images in a list, and each image is attached with some possible labels as an aid. The length of the suggestion list is 5 by default. Figure 2a shows three operations in the annotation task: label confirmation, selection, and typing. The users can produce a new label, and our classifier can generate a sub-classifier in the one-versus-all setting to realize the prediction of this new class. For the verification task, our system shows the selected images group by group based on their predicted labels. For each group, the users are required to point out the outliers, which can be classified in the next iterations. Since all the images in every group have the same category prediction, the outlier is the image whose label is different from the label of its group. As shown in Figure 2b, the three outliers in the red boxes are all ‘street’ images, which do not belong to the class ‘highway’. In practice, the users can make a subjective judgment based on their requirements. For example, when some people expect to combine the ‘highway’ and ‘street’ images into one category since they are both related to the concept ‘road’, the users can simply confirm the prediction in the situation shown in Figure 2b. If the prediction result contains a large amount of noise, indicating all the outliers is rather boring. Therefore, the verification process stops once the users choose three consecutive images as the noise of the group. All the unhandled images in this group are automatically treated as outliers.

**Online learning.** The training phase starts when human feedback is achieved. To match our iterative classification scenario, we design a weighted blending framework to predict the category label of the image. The framework is a combination of two classifiers. The first one is the SoftMax classifier layer of the deep model trained in the offline session, which remains constant during online classification. On the contrary, the second classifier is learned and updated by training new classified image data in an online manner, which represents the personalized intent of categorization. Since it requires a long-term process to train an online classifier, our blending framework helps to alleviate the cold-start problem.

**Label Prediction.** To reduce user effort, we use the similarity between the images to boost the output of the weighted blending framework. To this end, our system formulates the label prediction problem as an efficient conditional random field, where the nodes correspond to the unclassified images. To generate the graph structure of CRF, we create k−nearest neighbor graph based on the distance of the image features. The CRF optimization makes similar images have the same prediction, which leads to a more consolidated classification result.

## 4. Feature Learning

The traditional methods usually use the cross-entropy loss to train a deep model, which aims at minimizing the intra-class distance of the features. As these methods are targeting a fixed taxonomy, they are not suitable for personalized tasks. Another method is to train different models for different taxonomies. However, due to the diversity of the taxonomies in the personalized tasks, it is impractical to collect the training data pre-labeled with all the required taxonomies.

**Figure 3:**
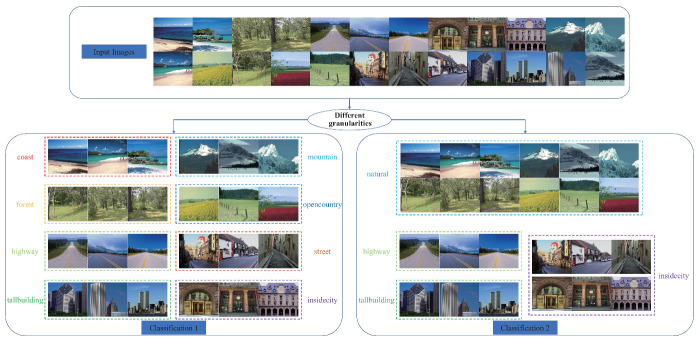
Given an image set, different classification results can be generated with different granularities.

Though the users’ preferences are diverse and unpredictable, they are usually different in the classification granularities between multiple users. especially, classification granularity is one of the multiple levels defined in the label hierarchy from WordNet. The is used to describe the coarse degree of the classification criterion. Figure 3 shows two classification results with different granularities of the same image set. Based on different granularities, the given set can be divided into four or eight subsets. Based on this observation, we propose a feature learning algorithm based on semantic embedding. This method makes the extracted feature maintain the semantic similarity between the label concepts of the images, instead of guaranteeing only the discrimination between any different classes of images. especially, we introduce a pre-trained GloVe model [7] to transform the original discrete category label into the continuous vector. Based on the label conversion, we can use the distance of two vectors to quantify the degree of semantic similarity between different categories. The degree of similarity is then encoded into the feature learning process by a log-ratio loss term. Meanwhile, the cross-entropy loss is also used to maintain the basic discrimination information. This leads to a compact feature descriptor that covers the discrimination information of multiple granularities.

Given a training image $I_{t}$, we indicate its category label as *c*. For each category *c* in the training set, we extract a *d*-dimensional semantic-embedding vector using the pre-trained GloVe model [7]

(1) $$v_{c}=f_{g}\left(c\right)$$

where $f_{g}$ is the pre-trained GloVe model, which is used to obtain vector representations for words. The goal of the label conversion is to realize the information enhancement of the image labels.

Based on this transformation, the distance between the category $c_{1}$ and $c_{2}$ can be measured by the Euclidean norm $∥v_{c_{1}}-v_{c_{2}}∥$. After the transformation, every training image $I_{t}$ has two labels: the discrete label *c* and the continuous label $v_{c}$.

To process the continuous label, inspired with the research in deep metric learning beyond binary supervision [9], we use a log-ratio loss to realize model learning with the continuous label $v_{c}$. Meanwhile, we also use the cross-entropy loss to handle the discrete label *c* as the standard classification loss.

**Loss Function.** We indicate the training image set as $T=\{I_{t_{i}},c_{i},v_{c_{i}}|i=1,...,N\}$, where $c_{i}$ and $v_{c_{i}}$ are the labels of the image $I_{t_{i}}$. The definition of the cross-entropy loss is followed as:

(2) $$l_{ce}\left(I_{t}\right)=H\left(c,f_{c}\left(I_{t}\right)\right)$$

where $f_{c}\left(\cdot \right)$ is the output of the classifier layer in the deep model, and H(·,·) is the cross-entropy between two distributions.

To learn with the continuous label, we introduce a log-ratio loss, which takes a triplet of an anchor $I_{a}$ and its two neighbors images $I_{i}$ and $I_{j}$ as input. This loss measures the difference between the ratio of the label distance and the ratio of the feature distance. Given the three images and their continuous labels $v_{c_{a}}$, $v_{c_{i}}$ and $v_{c_{j}}$, we define the log-ratio loss as

(3) $$l_{lr}\left(I_{a},I_{i},I_{j}\right)={\left\{log\frac{D(f_{f}\left(I_{a}\right),f_{f}\left(I_{i}\right))}{D(f_{f}\left(I_{a}\right),f_{f}\left(I_{j}\right))}-log\frac{D(v_{c_{a}},v_{c_{i}})}{D(v_{c_{a}},v_{c_{j}})}\right\}}^{2}$$

where $f_{f}\left(\cdot \right)$ is the output of the feature extractor in the deep model, D(·,·) is the squared Euclidean norm.

Hence, given a triplet $\left(I_{a},I_{i},I_{j}\right)$, the final loss function is defined as the sum of the cross-entropy loss and the log-ratio loss

(4) $$L\left(I_{a},I_{i},I_{j}\right)=l_{ce}\left(I_{a}\right)+l_{ce}\left(I_{i}\right)+l_{ce}\left(I_{j}\right)+l_{lr}\left(I_{a},I_{i},I_{j}\right)$$

**Training Data Generation.** Since the input of the loss function contains three images, we use a dense triplet mining method [9] to generate the training data. First, a mini-batch *B* of images is created by selecting an anchor $I_{a}$ and its k−nearest neighbors measured by label distance. To cover the whole training set, we expand the set *B* by randomly sampling some images from the remaining ones. We then choose every pair of images $\left(I_{i},I_{j}\right)$ in *B* excluding the anchor. The training triplets are finally generated by combining all the pairs with the anchor $I_{a}$. It is worth noting that in the log-ratio loss, we do not need the distance between the anchor and the positive one is smaller than that between the anchor and the negative one. The log-ratio loss only preserves the ratio of the label distance in the feature space. Thus, the effect is identical when swapping the image $I_{i}$ and $I_{j}$. To avoid duplication, we ignore the triplet $I_{a},I_{i},I_{j}$ when $D\left(v_{c_{a}},v_{c_{i}}\right)<D\left(v_{c_{a}},v_{c_{j}}\right)$. The sampling result T from *B* is finally given by

(5) $$T=\left\{\left(I_{a},I_{i},I_{j}\right)|D\left(v_{c_{a}},v_{c_{i}}\right)>D\left(v_{c_{a}},v_{c_{j}}\right),c_{i}\in B\setminus \left\{I_{a}\right\},c_{j}\in B\setminus \left\{I_{a}\right\}\right\}$$

When constructing *B*, we add our own constraint to make it applicable to our task: given the anchor $l_{a}$ with its category label *c*, all of its neighbors are generated from the images that have the labels other than *c* mandatorily. The motivation for introducing this constraint embraces two aspects. First, it avoids dividing by zero in Equation (3). Second, without this constraint, all the *k* nearest neighbors probably belong to the category *c*, since *k* is usually smaller than the number of the images labeled by *c*. However, we can find that when $I_{i}$ belongs to the category *c*, the ideal molecule should be zero in Equation (3), and the term $v_{c_{j}}$ has no effect on the loss. Thus, the degrees of label similarities cannot be provided, which makes the loss deteriorate into the normal classification loss.

After generating the training data, we optimize the deep network through back-propagation with stochastic gradient descent. We use ResNet50 [2] as the backbone network of our network. The network can be pre-trained based on ImageNet with the cross-entropy loss and then fine-tuned on a specific dataset with our loss function. In our experiment, we split the benchmark image set into two disjoint parts. One part is used for network fine-tuning, while the other part is used for online classification. Finally, we use the penultimate layer in the network as the feature representation during the online session.

**Figure 4:**
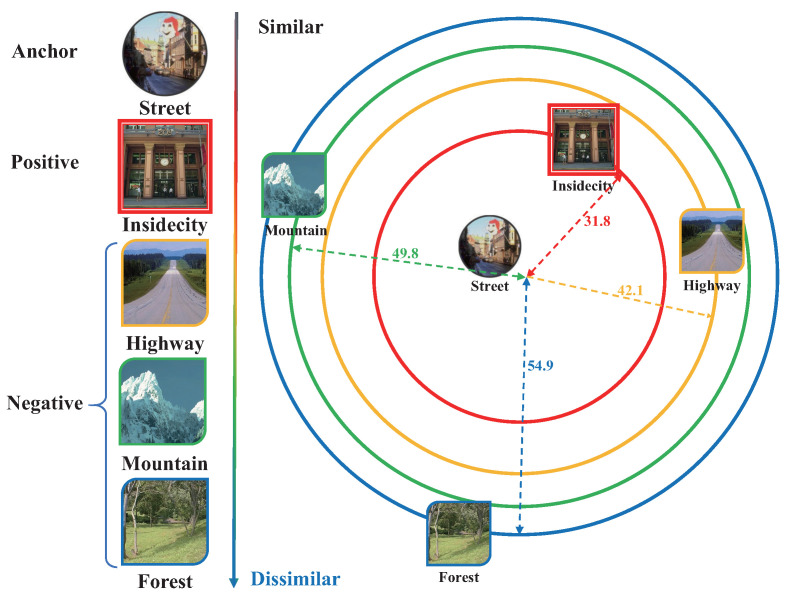
An illustration of our feature learning strategy. In this example, the anchor is a ’street’ image. The positive image is an ’insidecity’ image, since the similarity between ’street and ”insidecity’ is the highest. The other three images can be selected as the negative images. The number shows the label distance between different classes.

Figure 4 shows an example of our feature strategy. To simplify the description, we only select one positive image for the anchor ‘street’ image. Based on the label distance, the ‘insidecity’ image has the largest similarity, which is then selected as the positive image. To create the triplet, we randomly select another image from the remaining three images as the negative image. If more positive images are required to be sampled, we simply choose the images based on the label distance in descending order.

**Figure 5:**
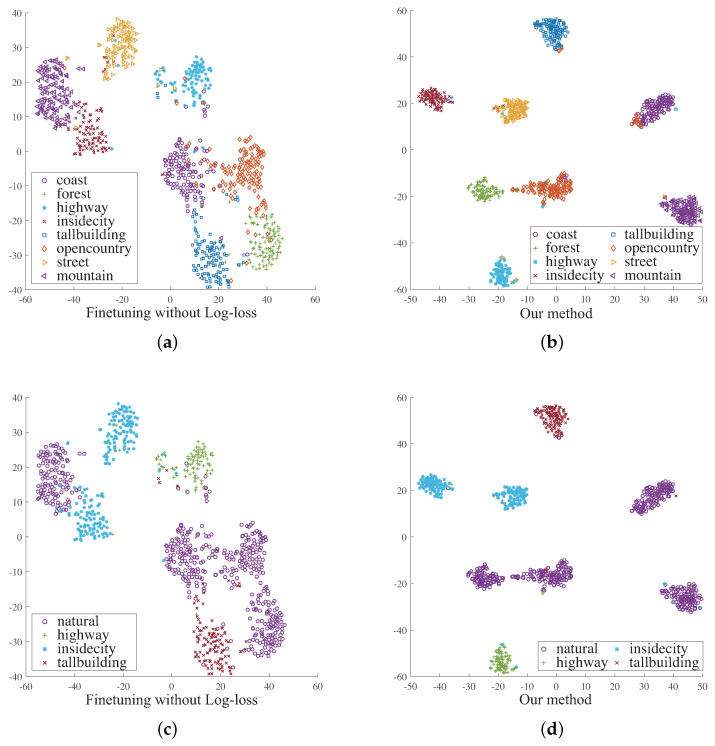
Different 2D projections of image features across two taxonomies. The features are generated by two different networks learned without log-loss term and with our loss function. (**a**): taxonomy 1 without log-loss term; (**b**): taxonomy 1 with our loss; (**c**): taxonomy 2 without log-loss term; (**d**): taxonomy 2 with our loss.

During the training, the network not only improves the discrimination of the learned feature, but also encourages that the ‘insidecity’ class is the nearest to the ‘street’ class. Such a feature distribution reduces the complexity of the classifier training when some users expect to put the ‘insidecity’ and ‘street’ images into one class. Figure 5 shows the advantage of introducing the log-ratio loss. In this figure, we compare two feature distributions of the same image set, where the features are generated from two networks learned by different objective functions. The first network is learned with the cross-entropy loss, while the second network is trained with the combination of the cross-entropy loss and the log-ratio loss. The extracted image features are then projected in 2D space using parametric *t*-SNE [28]. We also label the points in the 2D space based on two different taxonomies, which are consistent with the criterion shown as in Figure 3. As shown in Figure 5, the feature generated by the second network provides higher discrimination since the distances between different classes are more significant. Moreover, the second network can provide a more flexible feature representation. As shown on Figure 5c, it is difficult to find a simple classification boundary in the distribution, since the ‘natural’ class has a large overlap in the space between the class and the ‘insidecity’ class. In contrast, Figure 5d shows a distribution that is adaptive to such a change of classification granularities.

## 5. Annotation and Verification Selection

In this section, we introduce an active learning algorithm to realize image selection for annotation and verification. The input to the algorithm is the set of unclassified images denoted by $S_{m}$ when the *m*th iteration starts. The image selection algorithm is performed twice at the *m*th iteration: first to decide the optimal annotation set $A^{m*}$ and second, after the annotation task, to decide the optimal verification set $V^{m*}$. The set $A^{m*}$ and $V^{m*}$ are two disjoint subsets of the unclassified image set $S_{m}$. Inspired with active 3D region annotation method [22], we formulate image selection as a combinatorial optimization problem, and propose a classification efficiency model for the multi-class setting. especially, we define the optimization variable as the candidate annotation set $A^{m}$ and the candidate verification set $V^{m}$ respectively. To define the optimization objective, we first measure the number of classified images $N^{m}$ and the expended timing $T^{m}$ as the function of the optimization variable $A^{m}$ and $V^{m}$, and then use the ratio $N^{m}/T^{m}$ as the classification efficiency of the *m*th iteration. Accordingly, the optimal set $A^{m*}$ and $V^{m*}$ are the solution of the optimization problem, which has the maximum efficiency among all the candidate sets. In this section, we first detail the formulation of the efficiency model, then describe the efficiency optimization method for annotation and verification successively.

### 5.1. Efficiency Model

To realize the optimization, we should determine how the image number $N^{m}$ and the timing $T^{m}$ depend on the candidate set $A^{m}$ and $V^{m}$ quantitatively. As the previous image number $N^{m-1}$ and the previous timing $T^{m-1}$ are known, we only require to estimate the number of new classified images and the increased timing during *m*th iteration. However, since human input cannot be determined in advance, we leverage a probabilistic formulation to obtain the expected values of the terms.

First, we describe how to estimate the image number $N^{m}$ given the set $A^{m}$ and $V^{m}$. For *m*th iteration, both the annotation and verification tasks generate the new classified images. For the annotation, all the annotated images become the new classified images. For the verification, we count the new classifier images by excluding the outliers. Since we do not determine which images to be indicated as outliers, our system obtains the expected number of new classified images as the sum of the prediction confidences on all the images. Thus, we compute $N^{m}$ by

(6) $$N^{m}=N^{m-1}+\left|A^{m}\right|+\sum_{i=1}^{|V^{m}|}p_{i}^{p}$$

where $p_{i}^{p}$ is the probability of the user confirming the prediction of the *i*th image in $V^{m}$, which is the same with the prediction confidence.

Then, we detail how to estimate the timing $T^{m}$ with respect to the $A^{m}$ and $V^{m}$. To measure the timing $T^{m}$, we should estimate how long it would take to complete the annotation and verification by the users. However, the different operation requires different timing costs, for example, the confirming operation usually spends much time than the typing operation. This brings challenges to the estimation of the timing $T^{m}$, since we have no idea which operation will be taken by the users. To solve it, we first use the prediction confidence to obtain the probability of the user executing a certain operation, and then achieve the approximate timing cost as the weighted sum of the statistics timing of all the possible operations.

Given an image $x_{i}$, we denote its category prediction by the a sorted probability vector ${\vec{\eta }}_{i}=\left\{\eta_{i1},\eta_{i2},...,\eta_{il}\right\}\left(\eta_{i1}\geq \eta_{i2}\geq ...\geq \eta_{il}\right)$) (*l* is the number of existing classes), which is generated by sorting the output probability vector of the weighted blending framework in descending order (described in Section 6). $\eta_{ij}$ is the *j*th largest category probability. Based on the probability vector, we can achieve the probability of the user executing a certain operation. especially, for the annotation, we have three probabilities $p_{i}^{c}$, $p_{i}^{s}$ and $p_{i}^{t}$ corresponding to the label confirmation, selection and typing operation respectively. For verification, we have two probabilities $p_{i}^{p}$ and $p_{i}^{n}$ corresponding to confirmation and outlier indication. These five probabilities are computed by

(7) $$\begin{array}{cc} \; & p_{i}^{c}=\eta_{i1}\\ \; & p_{i}^{s}=\sum_{j=2}^{5}\eta_{ij}\\ \; & p_{i}^{t}=1-p_{i}^{c}-p_{i}^{s}\\ \; & p_{i}^{p}=\eta_{i1}\\ \; & p_{i}^{n}=1-p_{i}^{p} \end{array}$$

Given these five probabilities, we can compute the timing cost of annotating the *i*th image in $A^{m}$ as the weighted sum of several timing constants. The timing cost of verifying the *i*th image in $V^{m}$ can be achieved in the same way. Thus, the two timing costs $\tau_{i}^{A}$ and $\tau_{i}^{V}$ are

(8) $$\begin{array}{cc} \; & \tau_{i}^{A}=p_{i}^{c}\tau_{i}^{c}+p_{i}^{s}\tau_{i}^{s}+p_{i}^{t}\tau_{i}^{t}\\ \; & \tau_{i}^{V}=p_{i}^{p}\tau_{i}^{p}+p_{i}^{n}\tau_{i}^{n} \end{array}$$

where $\tau_{i}^{c}=1.5$ s, $\tau_{i}^{s}=4.0$ s and $\tau_{i}^{t}=5.0$ s are the times required to execute confirmation, selection and typing during the annotation task. $\tau_{i}^{p}=0.4$ s and $\tau_{i}^{n}=1.2$ s are the times of execute confirmation and outlier indication during the verification task. All the timing constants are estimated through experimental statistics.

The expected timing $T^{m}$ is therefore computed by adding the timing costs of all the images in the candidate set $A^{m}$ and $V^{m}$:

(9) $$T^{m}=T^{m-1}+\sum_{i=1}^{|A^{m}|}\tau_{i}^{A}+\sum_{i=1}^{|V^{m}|}\tau_{i}^{V}$$

**Figure 6:**
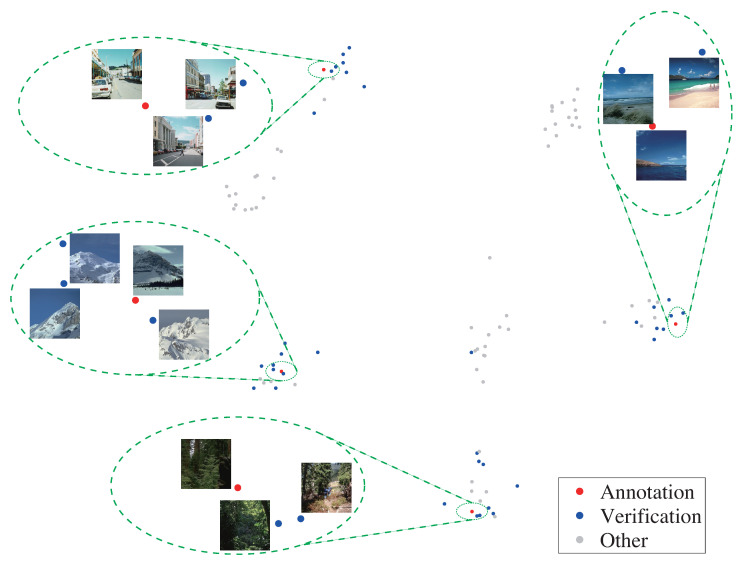
Our method iteratively selects an annotation set (in red) and a verification set (in blue) from the image collection (in gray). The annotation set covers the entire collection, and the verification set stays around the annotated images since the prediction has high confidence.

Finally, we can obtain the expected efficiency $E^{m}$ by $N^{m}/T^{m}$ as the optimization objective. Based on this objective function, we then use an optimization algorithm to select the optimal annotation and verification set by maximizing the efficiency. Figure 6 shows an example of the images selected to be annotated and verified at the first iteration, when classifying 100 scene images.

### 5.2. Annotation Selection

A challenge problem is that we cannot obtain the expected efficiency with only a candidate annotation set $A^{m}$, since the set $V^{m}$ should be given. However, it is impractical to use brute-force search for the best candidate verification set for the set $A^{m}$. Thus, we construct a suboptimal verification set $V^{m^{\prime }}$ to measure the expected efficiency $E^{m}$. To construct the set $V^{m^{\prime }}$, we first simulate the manual annotation process with a pseudo-labeling method. To realize the pseudo-labeling process, we confirm the top label of the suggest list for all the image of the set $A^{m}$. Based on the simulated result, we execute the online learning and label prediction. The verification selection is then used to create the set $V^{m^{\prime }}$, as described in Section 5.3.

Based on the suboptimal verification set $V^{m^{\prime }}$, we can then search the best annotation set $A^{m}$ by maximizing the efficiency: $A^{m}=\arg\max_{A}E^{m}\left(A,V^{m}\right)$. The size of the annotation set is fixed ($|A^{m}|=0.005N$). To speed up the search, we create a limited search set *D* ($\left|D|=2\right|A^{m}|$), which consists of several representative images of the unclassified image set $S_{m}$. These images are sampled based on the k−means clustering of the set $S_{m}$. Given the clustering result, we select the center image from every cluster as the representative.

We then employ beam search to iteratively search the subsets of the limited set *D*, and use the subset with the maximum efficiency as the optimal set $A^{m}$. especially, we maintain a set of suboptimal solutions $\Omega_{k}$ at iteration *k*, where the size of $\Omega_{k}$ is fixed as $|A^{m}|$. Each initial suboptimal solution is a singleton. Thus, to create $\Omega_{0}$, we construct |D| singletons as the candidate annotation sets, and choose $|A^{m}|$ singletons with the higher expected efficiency. At iteration k>0, each solution in $\Omega_{k-1}^{l}$ is expanded by adding one image in *D*. We then treat every expanded set as the annotation set to compute the efficiency, and reserve $|A^{m}|$ sets with higher efficiency among these expanded sets to create $\Omega_{k}$. Our system stops the searching when the size of each suboptimal solution of $\Omega_{k}$ is equal to $|A^{m}|$, and selects the best solution in $\Omega_{k}$ as the optimal annotation set.

### 5.3. Verification Selection

We use the sample selection algorithm [22] to perform verification selection. Since the annotation set $A^{m}$ is fixed, verification selection is rather simple. To create the optimal $V^{m}$, we first sort all the unclassified images with their prediction confidences in descending order, and then expand $V^{m}$ by adding one image successively in an iterative manner. When the efficiency $E^{m}$ stops rising, we can find the optimal verification set $V^{m}$.

## 6. Online Learning

The annotation and verification tasks produce some new classified images, which shows the personalized requirement. By using these classified images, we can train a customized classifier to capture the user’s intent of categorization. However, during the early stage, the performance of the classifier is not satisfactory. To resolve it, we design a weighted blending framework to combine a pre-trained SoftMax classifier (as described in Section 4) and an online soft confidence weighted (SCW) classifier [10], which is inspired with personalized image annotation [16]. This framework gathers the classification information from common taxonomy and personalized taxonomy, which helps to alleviate the cold-start problem.

To train the customized classifier $C_{u}$, we adopt the SCW classifier to learn from the new classified images, since it is suitable for an online multi-class learning situation. To support multi-class classification, a one-versus-all setting is used. especially, the SCW classifier $C_{u}$ consists of several binary classifiers, which are denoted by ${\left\{\left(\mu^{i},\Sigma^{i}\right)\right\}}_{i=1}^{M}$. $\mu^{i}$ and $\Sigma^{i}$ are the mean vector and covariance matrix of a multivariate Gaussian distribution. Initially, $\mu_{0}=0,\Sigma_{0}=I$. When predicting the category of a feature vector *x*, the SCW classifier checks every binary classifier and selects the class with the highest score based on the matching degree between the feature *x* and the Gaussian distribution. When training the SCW classifier, the classifier is updated one by one in an online manner without retraining the classified images in the previous iteration. Thus, we use only new classified images from the annotation and verification task in the current iteration as the training data. To perform classifier training, we sort all the new classified images randomly into a data stream, which is denoted by a sequence $\left\{\left(x_{t},y_{t}\right)\right\}$, where $x_{t}$ is the *i*th image represented by a feature vector, and $y_{t}$ is the label of the image. The SCW classifier is then updated by traversing the sequence. Given a training image $\left(x_{t},y_{t}\right)$, SCW updates the mean vector $\mu^{i}$ and the covariance matrix $\Sigma^{i}$ to obtain a new multivariate Gaussian distribution. The updated distribution makes the score of the correct class to be higher than the largest score of the other classes above a certain threshold. More detail can be found in [10].

In the annotation task, the users might use a new label $y^{\prime }$ to achieve the label typing operation. This means a new class may appear at any time. The SCW classifier naturally supports this kind of dynamic environment, since it adopts the one-versus-all setting. Given a training image represented by the feature *x* with a new label $y^{\prime }$, we add a binary classifier $\left(\mu^{\prime },\Sigma^{\prime }\right)$ to the set of binary classifiers in the SCW classifier $C_{u}$, where $\mu^{\prime }$ and $\Sigma^{\prime }$ are initialized to the feature *x* and the identity matrix I respectively. The mean vector $\mu^{\prime }$ and the covariance matrix $\Sigma^{\prime }$ can be updated when more training images labeled by $y^{\prime }$ emerge. With the accumulation of the training images, the multivariate Gaussian distributions of the customized classifier $C_{u}$ are more consistent with the desired partition of the high-dimensional feature space, which is captured from the interactive annotation and verification task gradually. During the prediction session, if the binary classifier $\{\left(\mu^{\prime },\Sigma^{\prime }\right\}$ has the largest score among all the binary classifiers, the label of the image is predicted as $y^{\prime }$. It is worth noting that the user can use any free label as an alternative to the label $y^{\prime }$, since the unique function of the label is to determine which images belong to the same category. especially, the typing label $y^{\prime }$ does not affect the feature extraction process, since the pre-trained deep network is not updated during the online classification. Meanwhile, the new label $y^{\prime }$ needs not to be constrained in the GloVe model, since the semantic-embedding vector $v_{c}$ derived from the GloVe model is not used in the feature extraction session.

The weighted blending framework takes the learned SCW classifier $C_{u}$ and the pre-trained SoftMax classifier $C_{m}$ as two components. Given the feature of an image *x*, the weighted blending framework generates a probability vector $\vec{\varepsilon }=\left\{\varepsilon_{1},\varepsilon_{2},...,\varepsilon_{l}\right\}$, where *l* is the number of existing classes, and $\varepsilon_{i}$ is the probability of the image *x* belonging to the *i*th category $c_{i}$. The vector $\vec{\varepsilon }$ is generated by combining the predictions of the classifiers $C_{m}$ and $C_{u}$ with a weighting factor *w*:

(10) $$\varepsilon_{i}=max\left[P\left\{C_{u}\left(x\right)=c_{i}\right\},w\cdot P\left\{C_{m}\left(x\right)=c_{i}\right\}\right]$$

where $P\{C_{u}\left(x\right)=c_{i}\}$ and $P\{C_{m}\left(x\right)=c_{i}\}$ are the probability distributions, which are corresponding to the class $c_{i}$. They are from the prediction result of the image *x* by using the classifier $C_{u}$ and $C_{m}$ respectively. To determine the value of the weighting factor *w*, we simulate the interactive classification process and choose the optimal value based on average efficiency maximization. According to the experiment, the weighting factor *w* is fixed as 1.0.

## 7. Label Prediction

In this section, we detail our CRF framework for label prediction. The goal of this framework is to achieve a more satisfactory classification of the unclassified images, which are used to generate the suggestion list in the annotation task and the images to be verified for interactive verification. The input of the framework is the set *X* of all the unclassified images. By the weighted blending framework, we can obtain the probability vector ${\vec{\varepsilon }}_{i}$ for every unclassified image $x_{i}\in X$. According to these probability vectors ${\vec{\varepsilon }}_{i}$, the CRF framework uses the similarity between the two unclassified images to generate a more precise classification result.

Our motivation is that the similarity can provide supplementary information to help classify data since similar instances are more likely to share the same class label [29]. Accordingly, we design a CRF framework to combine the probability vectors ${\vec{\varepsilon }}_{i}$ and the similarity between different images. especially, we first encode the probability vectors into the unary potentials, and then use the similarity as the pairwise potentials to constrain the final classification result. We indicate the optimal classification result of the unclassified image set *X* as $L=\{c_{1},c_{2},...,c_{\left|X\right|}\}$, where $c_{i}$ is the class label of the image $x_{i}\in X$. First, the unary potentials $\phi_{i}\left(c_{i}\right)$ is defined as follows:

(11) $$\phi_{i}\left(c_{i}\right)=-log\left(\varepsilon_{i}\left(c_{i}\right)\right)$$

where $\varepsilon_{i}\left(c_{i}\right)$ is the probability of the image $x_{i}$ belonging to the category $c_{i}$. Thus, minimizing this potential makes the optimal label $c_{i}$ consistent with the corresponding prediction of the probability vector ${\vec{\varepsilon }}_{i}$.

Next, we define the pairwise potentials of CRF framework based on a k−NN graph X by treating all the unclassified images as the node. The edge of the k−NN graph X is created according to the feature distance (k=10). To encourage similar images to have a similar prediction, we define the pairwise potentials $\varphi_{ij}\left(c_{i},c_{j}\right)$ as follows:

(12) $$\varphi_{ij}\left(c_{i},c_{j}\right)=\mu \left(c_{i},c_{j}\right)\alpha \left(x_{i},x_{j}\right)$$

where $\mu (c_{i},c_{j})$ is the label compatibility term, if $c_{i}=c_{j}$, $\mu (c_{i},c_{j})=0$, otherwise, $\mu (c_{i},c_{j})=1$. The weight $\alpha (x_{i},x_{j})$ is defined in terms of the feature similarity between $x_{i}$ and $x_{j}$, which shows the contribution of the pairwise term to the objective function. As shown in the function, the pairwise potentials can be minimized by making all the similar images have the same prediction.

Finally, we define the objective function of CRF framework by combining the unary potentials $\phi_{i}\left(c_{i}\right)$ and the pairwise potentials $\varphi_{ij}\left(c_{i},c_{j}\right)$ as follows:

(13) $$\min_{L}\sum_{i=1}^{\left|X\right|}\phi_{i}\left(c_{i}\right)+\sum_{(x_{i},x_{j})\in X}\varphi_{ij}\left(c_{i},c_{j}\right)$$

where $(x_{i},x_{j})\in X$ means $x_{i}$ and $x_{j}$ are connected in the k−NN graph X. By using TRW-S [30], we minimize Objective (13) to achieve a more reliable category prediction, which is used as the classification hypotheses for online annotation and verification task.

## 8. Results

In this section, we evaluate our interactive system by comparing it against previous work and validating our technical choices. Our experiment environment is Intel(R) Core(TM) i7-8700 3.20 GHz with 32 GB of memory.

### 8.1. Experimental Setup

The method is evaluated on 4 image sets: Scene8 dataset [31], UIUC-Sports event dataset [32], PASCAL Visual Object Classes 2007 (VOC2007) [33], Caltech101 [34]. For each image set, we split the whole set into the training set and the evaluation set. The training set is used for CNN fine-tuning during feature learning, which achieves the network for feature extraction. To realize the training, we use the existing labels of these datasets as the discrete labels of our feature learning method.

To show the effectiveness of our method, we perform a real-world user study on the Scene8 dataset to evaluate our method with the comparison of the previous work. To analysis the effect of different design, we first reclassify the evaluation set based on multiple criteria as the personalized categorization, and then measure the interactive rates across different taxonomies.

**Scene8 dataset.** Scene8 dataset contains 8 outdoor scene categories. There are 2688 color images, 256 × 256 pixels. To realize our method, 1828 images are randomly sampled for CNN fine-tuning, and the other 860 images are used as the evaluation set. To reclassify the evaluation set, some fine-grained classes are combined into a superclass, as shown in Table 1. Totally, there are five different classification results for Scene8 dataset. Each line shows one classification result. For each criterion, if the labels do not appear in the corresponding line, it means that they remain unchanged. For example, for the first criterion in Table 1, we only combine the ‘opencountry’ and ‘forest’ images in Scene8 into a new group with the label ‘opencountry’, while keeping the categories of all the other images invariant. Thus, the class number is 7. Remarkably, some combinations do not match the label hierarchy implied in the GloVe model perfectly. For example, based on the semantic-embedding distance between different concepts by the GloVe model, the nearest concept to ‘forest’ is ‘mountain’. However, we prefer to combine ‘forest’ and ‘opencountry’ into one class, as shown in Table 1. The following experiment described in Section 8.5 shows that our system is still efficient when using such a taxonomy.

**Event dataset**. The dataset contains 8 sports event categories. Totally, there are 1579 images. Among these images, 789 images are randomly sampled based on the category distribution CNN fine-tuning, and the remaining 790 images are used as the evaluation set. We also generate 5 taxonomies by combining some fine-grained tags into one group. The results are shown in Table 2.

**VOC2007**. In total, there are 9963 images, which are classified into 20 categories. The images are from several visual object classes in the real world. The data has been split into the training and testing set with the same size. Some of the images have multiple labels. As our classification task does not consider the multi-label classification application, we remove them from the training and testing set. Finally, we use 2808 images CNN fine-tuning, and randomly choose 900 images as the evaluation set. The evaluation set is also regrouped according to the hierarchical structure of the semantic concept, as shown in Table 3.

**Caltech101**. Caltech101 consists of images of objects belonging to 101 classes. Each image is labeled with a single category label, and the typical edge lengths are 200–300 pixels. Each category contains roughly 40 to 800 images, totally around 9000 images. To realize feature learning, we split the dataset into 90% for CNN fine-tuning and 10% for evaluation based on category distribution. Finally, the size of the training and evaluation set is 7955 and 722, respectively. Similar to the other three datasets, we also generate 5 taxonomies to reclassify the evaluation set, as shown in Table 4.

**Evaluation metrics**. The following experiments contain two types: real-world experiment and simulation experiment. The user-study experiment in Section 8.3 belongs to the real-world experiment, which asks the user to perform the classification task interactively. We then record the time of all the operations and extract the following metrics from the real record: the whole interactive timing and the number of each type of operation. Based on the real-world task, we can also estimate all the timing constants, which mean the interactive timing of each operation per one image. These timing constants can be used to achieve the approximate timing cost for comparison in the simulation experiment.

In the simulation experiment, we simulate the user’s operation based on the taxonomy, since the label of each image is known. After classifying all the images, we record the number of each type of operation. These numbers are not affected by subjective factors, since the taxonomy is definitive. We can then use the timing constants obtained from the user study to approximate the whole invested time. Finally, the efficiency ξ is defined as the ratio between the number of classified images and time investment when finishing the classification. The simulation experiment will use the efficiency ξ as the evaluation metric.

### 8.2. The Evaluation of Annotation Selection

**Figure 7:**
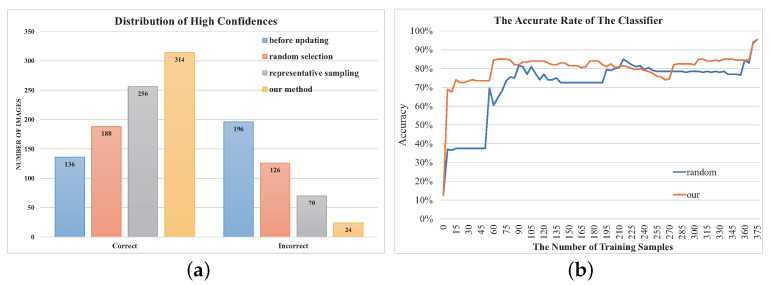
The Evaluation of Annotation Selection. (**a**): The distribution of high prediction confidences (more than 0.9). (**b**): The performance of the classifier with more training samples.

To compare our annotation selection, we use the testing 860 images from Scene8 as our evaluation set. The original label is used as the taxonomy. We choose the following two baseline methods. The first method is the random selection method (ran.), where the annotation set is created by randomly sampling some images from the unclassified image set. The second method is the representative sampling method (rep.) [35], which relies on the *k*-means clustering method and an initial classifier. These methods select different images as the annotation set. To compare the effect of these annotation sets, different classifiers are learned from the annotation sets. We then use a uniform test set to compare these classifiers’ performance. The test set consists of all the unlabeled images excluding the images in the three annotation sets. To compare with the representative sampling method, we choose the classifier learned at the second iteration. To make a fair comparison, we select the same annotation set for the first iteration all by our method. As shown in Table 5, our approach achieves the best accuracy rate. In addition, we investigate the prediction with high confidence (more than 0.9) of the three classifiers. We expect that there are more correct predictions when the confidence is high, since it ensures the low number of outliers during the verification task. As shown in Figure 7a, our method has more correct predictions and less incorrect ones than other methods among these predictions with high confidence. Meanwhile, the computation time of the selection is no more than 3 s, which shows that our method can support real-time interaction.

To show the relationship between the performance of the classifier and the number of training samples, we select 200 images randomly from the 860 images as the test set, and use our method to classify the 660 images based on the original taxonomy. We track the performance of the SCW classifier alone during our iterative classification process, since the performance of the SoftMax classifier is unchanged. When the classifier is updated, we test the accurate rate of the classifier on the test set. To compare with previous methods [6], we also record the performance of the classifier when the 660 images are trained in a random sequence. As shown in Figure 7b, our method can achieve a faster growth rate of the accuracy curve. With only 15 and 60 samples, the accuracy can reach 70% and 80% by our method. In contrast, the random method requires more samples, which are 60 and 90 respectively.

### 8.3. Comparison to Alternative Methods

In this section, we perform the use study to compare with the following two works: interactive streaming image classification (ISIC) [6] and PicMarker [5]. Meanwhile, we also compare the performance of our method with the conference version of this work (2018active) [11]. To compare the efficiency, we select the two image sets from Scene8 as the evaluation sets, which are used for the evaluation of ISIC and PicMarker, respectively. In the user studies, we recruited some users to complete the classification task by different systems according to their intent of categorization. For the two use studies, there were a total of 18 participants ranging in age from 18 to 35, including 8 females and 10 males. Most of them are the students at a university from a wide range of departments. All of them were heavy users of smartphones and computers, and had a lot of experience in taking and organizing photos. Thus, they did not feel difficult when identifying the content in the image, though they had not used our system before. Before the experiment, we showed a simple example for them, which classified 80 images with about three iterations. After that we asked them to use our system to complete their classification tasks without showing them the images in advance.

**Figure 8:**
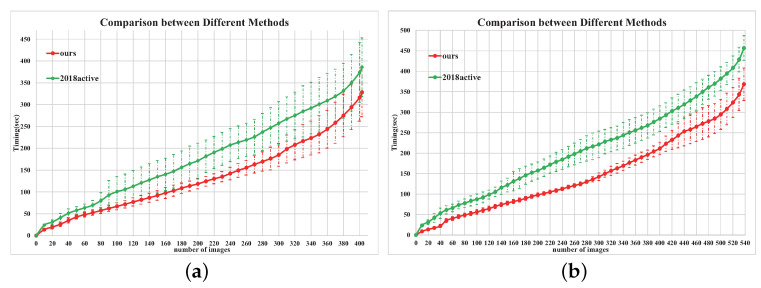
This figure shows the mean of expended timing with standard deviation(y-axis) as users classifying more images (x-axis). (**a**): the first image set; (**b**): the second image set.

During the classification process, the interactive process and the invested timing are recorded for comparison. For the first image set, we ask 12 users to classify 400 images with our system and our preliminary version. Figure 8a shows that the average of the user effort required by our method is less than that of the conference version—i.e., the curve of our system is lower. Meanwhile, the average timing of our method is 328.3 s. ISIC [6] reports the average timing of finishing the classification of the same 400 images, which is 656.3 s. In the user study of the conference version [11], the average timing is 385.8 s. Thus, for the first user study, our method is 2.0 times faster than ISIC, and 1.2 times faster than the conference version. For the second image set, we ask 6 users to classify 538 images by our system and our preliminary version. The efficiency comparison with the conference version is shown in Figure 8b, and the average timing of our method is 368.3 s. According to the user study in PicMarker [5], the average timing for classifying the same images is 561 s, which is 1.5 times longer than our method. Meanwhile, the average timing of the preliminary version is 456.7 s, which costs 1.2 times longer than ours. To summarize, our method can save more user effort than the previous works significantly.

**Figure 9:**
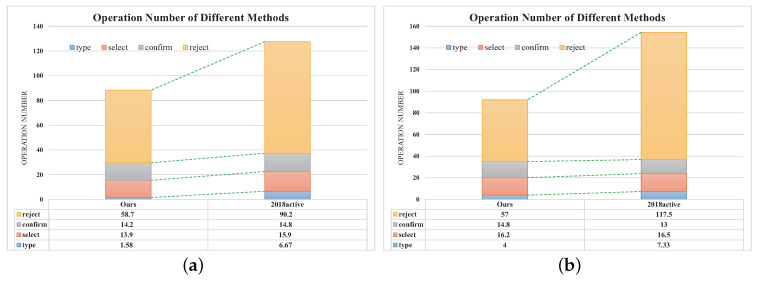
The comparison of different methods. This figure illustrates the average number of every operation required by our method and 2018active. (**a**): the first image set; (**b**): the second image set.

**Further Analysis**. To show the advantages of our method, we analyze and compare the interaction logs of our method and the conference version. especially, we focus on the number of typing, selecting, confirming, and negative rejecting operations. As shown in Figure 9, our system requires much less typing and rejecting operations than the conference version (2018active). The reasons include two aspects. On the one hand, the extracted feature is more powerful and adaptive for various taxonomies, which improves the accuracy of the classifier significantly. Therefore, the number of rejecting operations is reduced dramatically. On the other hand, we use the weighted blending framework to generate classification hypotheses. The pre-trained classifier can output many category labels in the suggestion list at the early stage. However, the conference version only uses the online learned classifier for prediction, which is learned from scratch. This leads to the cold-start situation, and the users are required to type the labels when a new class emerges. Thus, the conference version requires much more typing operation than our method.

**Timing Constants Estimation**. From the user study, we estimate all the timing constants used in our efficiency model, including the time required to execute confirmation, selection, and typing during annotation, and the time required to execute confirmation and outlier indication during verification. To estimate these timing constants, we construct a fitting model from the interactive log, which is solved by the least square method. The final timings are: $\tau_{i}^{c}=1.5$ s, $\tau_{i}^{s}=4.0$ s, $\tau_{i}^{t}=5.0$ s, $\tau_{i}^{p}=0.4$ s and $\tau_{i}^{n}=1.2$ s.

### 8.4. The Effect of the Weighting Factor w

The parameter *w* in the Equation (10) reflects the degree of personalization, which affects the performance of the automatic prediction. To evaluate the influence, this experiment executes our approach to the Scene8 dataset with different weighting factors, which ranges from 0.5 to 1.0. The sampling interval is set as 0.05, thus we generate 11 values. We use the evaluation set of Scene8 with the original ground truth and the other 5 criteria defined in Table 1. We compute the classification efficiency ξ across all the criteria. As shown in Table 6, the optimal value of the weighting factor is 1.0, as its average efficiency is highest, and the standard deviation is smallest. We expect the idea standard deviation to be small, since the interactive rate of the personalized classification system cannot be very sensitive to the diversity of the taxonomies.

### 8.5. Ablation Study

To reveal the effect of our technical choices, such as semantic embedding and active learning, we do some ablation studies to demonstrate their effectiveness. especially, we create some variants of our method by removing some novel elements as follows:

- **No log-ratio loss**—We only use the cross-entropy loss to train the deep network with semantic embedding.
- **No feature learning**—We represent each image by the ResNet50 network pre-trained on ImageNet.
- **No active selection**—We do not execute our annotation set algorithm and create the set with the same size by random sampling.
- **No verification**—The users are asked to perform the annotation task only.
- **No weighted blending**—We only use the online learned classifier for automatic prediction without blending the pre-trained SoftMax classifier.
- **No CRF**—We only use the weighted blending framework to predict the label of the unclassified image.
- **No verification stopping**—We do not check whether there are three consecutive outliers during the verification.

The ablation studies are performed on the four image sets. During the experiment, the annotation and verification tasks are performed automatically with a computer program given different taxonomies. To compare these methods, we compute the classification efficiency ξ as the evaluation metric.

**Figure 10:**
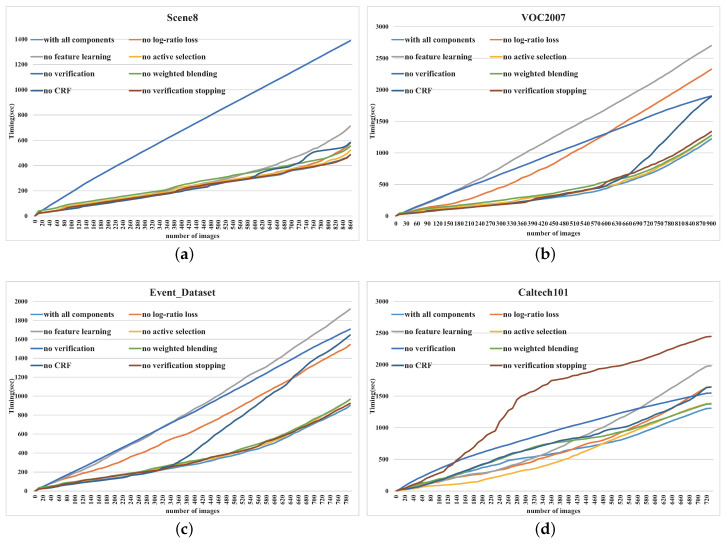
The comparison of different methods on the evaluation set. This figure illustrates the average of the required timing (y-axis) as people classifying more images (x-axis). (**a**): Scene8; (**b**): VOC2007; (**c**): Event Dataset; (**d**): Caltech101.

**Scene8**. We use the ground-truth criterion and the 5 criteria defined in Table 1 as multiple classification results, and run all the methods across multiple taxonomies. Table 7 shows the resulting efficiency ξ. As shown in Table 7, our method achieves impressive performance improvement compared with other methods, except the variant that disables verification stopping. The reason is that there are very few outliers in each group during the verification process in this experiment. Thus, the verification stopping mechanism does not work. As shown in Figure 10a, our method provides the fastest timing to task completion. Among all the technical elements, combining the verification step, and pre-training a deep model play the most significant role.

**VOC2007**. Similar to the setting of the experiment on Scene8, we run all the methods across multiple taxonomies, including the original criterion and the 5 criteria defined in Table 3. We then record the interactive log to compute the resulting efficiency ξ. Table 8 shows the efficiencies of all the methods, and our method achieves the state of art across all the criteria. Figure 10b shows a comparison between the methods. As shown in this figure, incorporating the log-ratio loss, and pre-training a deep model play the most significant role.

**Event Dataset**. Again, we run all the methods across the original taxonomy and the 5 reasonable taxonomies defined in Table 2. As shown in Table 9, our method achieves the state of art almost across all the criteria. Figure 10c shows the comparison between the methods. As shown in this figure, incorporating the log-ratio loss, CRF, the verification process, and pre-training a deep model play the most significant role.

**Caltech101**. Table 10 shows the classification efficiencies of all the methods across all the criteria, including the original criterion and the 5 criteria defined in Table 4. From the table, we can see that our method achieves the state of art almost across all the criteria. Figure 10d shows the comparison between the methods. As shown in this figure, incorporating verification stopping mechanism, and pre-training a deep model play the most significant role. It is worth noting that introducing the verification stopping mechanism is the most remarkable factor to improve efficiency, due to the low performance of the automatic prediction. The reason is that classifying Caltech101 is more challenging than the other images, since the size of each category in Caltech101 is much less than that in the other image sets. This brings some difficulties to train a reliable classifier as the training images are not enough.

**Summary**. After collecting all the statistical data across the 4 image sets, we summarize the contribution degree of each component to the efficiency improvement. To compare the contribution degree of the component, we compute the ratio between the classification efficiency ξ of the variant with the component disabled and that of the method with all the components. Thus, the lower the value of the ratio, the more the contribution degree of the component. Table 11 shows the results. From the table, we can find that introducing the verification process is the most important component on average, since it provides a high-throughput way for interactive classification. However, when the performance of the automatic prediction is poor (e.g., Caltech101), the effect of this component is not pronounced. Instead, the log-ratio loss, feature learning, and weighted blending components have an evident and relatively stable effect across the classification tasks of all the 4 image sets.

## 9. Conclusions

In this paper, we propose a novel interactive system for personalized image classification, which integrates semantic embedding, active learning, online learning into a unique pipeline. To extract the image feature for personalized classification, we propose a feature learning algorithm by using semantic embedding, which allows the degree of similarity in the semantic-embedding label space to be preserved in the feature space. During the online classification process, we introduce an active learning algorithm to realize the annotation and verification selection, which can improve the efficiency of interactive classification. Our experiments have shown that our system provides a flexible and efficient tool for user-adaptive image classification. However, our system focuses on personalized classification with different granularities. This limits the application, since the users can also classify images from different perspectives or attention. In the future, we will try to introduce the attention mechanism into our framework. Moreover, it is worth conduct our method for more complex tasks, such as image segmentation and image synthesis. 

## Figures and Tables

**Table 1:** Different classification results of Scene8 Dataset.

Criterion	Class Number	The Combination of Tags
1	7	opencountry ←{opencountry, forest} *
2	7	insidecity←{insidecity, street}
3	6	opencountry ←{opencountry, forest}, insidecity←{insidecity, street}
4	4	natural ←{coast, forest, mountain, opencountry}, insidecity←{insidecity, street}
5	2	natural ←{coast, forest, mountain, opencountry}, manmade←{insidecity, street, highway, tallbuilding}

* ‘opencountry ←{opencountry, forest}’ means we combine ‘opencountry’ and ‘forest’ into one category ‘opencountry’.

**Table 2:** Different classification results of Event Dataset.

Criterion	Class Number	The Combination of Tags
1	6	ball ←{bocce, croquet, polo}
2	5	ball←{bocce, croquet, polo, badminton}
3	7	rowing ←{sailing, rowing}
4	4	ball←{bocce, croquet, polo, badminton}, rowing ←{sailing, rowing}
5	2	ball←{bocce, croquet, polo, badminton}, others←{sailing, rowing, rock-climbing, snowboarding}

**Table 3:** Different classification results of VOC2007.

Criterion	Class Number	The Combination of Tags
1	16	mammal ←{cat, cow, dog, horse, sheep}
2	14	vehicle←{airplane, bicycle, boat, bus, car, motorbike, train}
3	17	furniture ←{chair, dining table, potted plant, sofa}
4	7	mammal ←{cat, cow, dog, horse, sheep}, vehicle←{airplane, bicycle, boat, bus, car, motorbike, train}, furniture ←{chair, dining table, potted plant, sofa}
5	4	tool ←{airplane, bicycle, boat, bus, car, motorbike, train, bottle}, animal←{cat, cow, dog, horse, sheep, bird}, furniture ←{chair, dining table, potted plant, sofa, tv/monitor}

**Table 4:** Different classification results of Caltech101.

Criterion	Class Number	The Combination of Tags
1	91	instruments ←{accordion, euphonium, guitar, mandolin, metronome, piano, saxophone}, transportation←{motorbikes, airplanes, car side, helicopter}, face←{face, face easy}
2	94	transportation←{motorbikes, airplanes, car side, helicopter}, ship←{ferry, ketch, schooner}, chair←{chair, wheelchair, windsor chair}
3	89	insect ←{ant, butterfly, dragonfly, mayfly, tick}, dinosaur←{brontosaurus, stegosaurus}, beast←{leopards, cougar body, cougar face}, crocodile←{crocodile, crocodile head}, bird←{emu, flamingo, flamingo head, ibis, pigeon}
4	95	products←{bass, crab, crayfish, lobster}, flower ←{lotus, sunflower, water lilly}, tower←{minaret, pagoda}
5	69	instruments ←{accordion, euphonium, guitar, mandolin, metronome, piano, saxophone}, transportation←{motorbikes, airplanes, car side, helicopter}, face←{face, face easy}, ship←{ferry, ketch, schooner}, chair←{chair, wheelchair, windsor chair}, tower←{minaret, pagoda}, dinosaur←{brontosaurus, stegosaurus}, insect ←{ant, butterfly, dragonfly, mayfly, tick}, beast←{leopards, cougar body, cougar face}, bird←{emu, flamingo, flamingo head, ibis, pigeon}, products←{bass, crab, crayfish, lobster}, flower ←{lotus, sunflower, water lilly}, crocodile←{crocodile, crocodile head}

**Table 5:** The accurate rates of different training data. The best performance indicators are marked as bold.

Method	Initial	Ran.	Rep.	Ours
accuracy rate	44.7%	58.8%	78.9%	**92.8%**

**Table 6:** The classification efficiency with different weighting factors. The best performance indicators of all the taxonomies are marked as bold.

*w*	0.5	0.55	0.6	0.65	0.7	0.75	0.8	0.85	0.9	0.95	1.0
ori.	1.48	1.46	1.46	1.48	1.55	1.48	1.49	1.53	1.55	1.52	**1.64**
cri. 1	1.61	1.62	1.61	1.61	1.59	1.59	1.65	1.62	1.69	1.59	**1.73**
cri. 2	1.62	1.44	1.60	1.49	1.66	1.50	1.62	1.62	1.53	1.64	**1.67**
cri. 3	1.64	1.62	1.62	1.61	1.62	1.53	1.61	1.68	1.62	1.60	**1.75**
cri. 4	1.83	1.83	1.84	1.84	1.84	1.84	**1.92**	1.87	1.87	1.90	1.86
cri. 5	2.02	2.02	2.02	2.02	2.02	2.02	2.02	2.02	2.02	2.02	**2.05**
$\bar{\xi }$ ± *s*	1.70 ± 0.18	1.66 ± 0.20	1.69 ± 0.19	1.67 ± 0.19	1.71 ± 0.16	1.66 ± 0.20	1.72 ± 0.19	1.72 ± 0.17	1.71 ± 0.18	1.71 ± 0.18	**1.78** ± **0.14**

**Table 7:** Comparison of different variants of our method on Scene8. The best performance indicators of all the taxonomies are marked as bold.

	ori.	cri. 1	cri. 2	cri. 3	cri. 4	cri. 5	$\bar{\xi }$ ± *s*
with all components	1.64	**1.73**	**1.67**	**1.75**	1.86	**2.05**	**1.78** ± **0.14**
No log-ratio loss	1.44	1.43	1.38	1.36	1.66	1.76	1.50 ± 0.15
No feature learning	1.08	1.13	1.10	1.11	1.44	1.54	1.23 ± 0.18
No active selection	1.51	1.57	1.54	1.65	1.74	1.95	1.66 ± 0.15
No verification	0.61	0.61	0.61	0.62	0.63	0.63	0.62 ± 0.01
No weighted blending	1.12	1.39	1.33	1.46	1.92	2.03	1.54 ± 0.32
No CRF	1.38	1.40	1.39	1.54	1.84	1.96	1.58 ± 0.23
No verification stopping	**1.67**	1.69	1.65	1.69	**1.95**	**2.05**	**1.78** ± **0.15**

**Table 8:** Comparison of different variants of our method on VOC2007. The best performance indicators of all the taxonomies are marked as bold.

	ori.	cri. 1	cri. 2	cri. 3	cri. 4	cri. 5	$\bar{\xi }$ ± *s*
with all components	**0.59**	**0.68**	**0.73**	**0.63**	**0.90**	**1.12**	**0.77** ± **0.19**
No log-ratio loss	0.30	0.35	0.39	0.30	0.50	0.71	0.42 ± 0.14
No feature learning	0.27	0.30	0.33	0.28	0.40	0.54	0.35 ± 0.09
No active selection	0.58	0.66	0.69	0.62	0.85	1.01	0.73 ± 0.15
No verification	0.45	0.47	0.47	0.45	0.48	0.53	0.47 ± 0.03
No weighted blending	0.32	0.43	0.48	0.35	0.81	0.99	0.56 ± 0.25
No CRF	0.56	0.63	0.69	0.60	0.89	1.09	0.74 ± 0.19
No verification stopping	0.53	0.65	0.62	0.55	0.89	1.09	0.72 ± 0.20

**Table 9:** Comparison of different variants of our method on Event Dataset. The best performance indicators of all the taxonomies are marked as bold.

	ori.	cri. 1	cri. 2	cri. 3	cri. 4	cri. 5	$\bar{\xi }$ ± *s*
with all components	0.66	**0.85**	**0.88**	**0.73**	**1.06**	**1.42**	**0.93** ± **0.25**
No log-ratio loss	0.35	0.50	0.54	0.39	0.64	1.14	0.59 ± 0.26
No feature learning	0.29	0.39	0.43	0.33	0.47	0.92	0.47 ± 0.21
No active selection	0.63	0.79	0.85	0.68	0.93	1.31	0.87 ± 0.22
No verification	0.44	0.45	0.46	0.44	0.48	0.52	0.46 ± 0.03
No weighted blending	0.31	0.44	0.63	0.33	0.65	1.14	0.58 ± 0.28
No CRF	0.61	0.79	0.85	0.66	1.02	1.34	0.88 ± 0.25
No verification stopping	**0.67**	0.83	0.83	0.72	**1.06**	1.30	0.90 ± 0.22

**Table 10:** Comparison of different variants of our method on Caltech101. The best performance indicators of all the taxonomies are marked as bold.

	ori.	cri. 1	cri. 2	cri. 3	cri. 4	cri. 5	$\bar{\xi }$ ± *s*
with all components	**0.50**	**0.54**	**0.59**	**0.56**	**0.54**	**0.59**	**0.55** ± **0.03**
No log-ratio loss	0.45	0.43	0.47	0.43	0.43	0.43	0.44 ± 0.01
No feature learning	0.38	0.38	0.36	0.34	0.37	0.37	0.37 ± 0.01
No active selection	0.49	0.52	0.51	0.53	0.51	0.58	0.52 ± 0.03
No verification	0.46	0.47	0.46	0.47	0.46	0.48	0.47 ± 0.01
No weighted blending	0.38	0.49	0.40	0.43	0.44	0.53	0.45 ± 0.05
No CRF	0.49	0.53	0.53	0.53	0.52	0.56	0.53 ± 0.02
No verification stopping	0.26	0.24	0.32	0.32	0.29	0.40	0.30 ± 0.05

**Table 11:** The summarize of the contribution degree of each components. The best performance indicators of all the image sets are marked as bold.

	Scene8	VOC2007	Event Dataset	Caltech101	Average
No log-ratio loss	84.23%	54.87%	63.63%	79.23%	70.49%
No feature learning	69.03%	**45.64%**	50.55%	65.95%	57.79%
No active selection	93.03%	94.83%	92.89%	94.43%	93.79%
No verification	**34.71%**	61.37%	**49.77%**	84.18%	**57.51%**
No weighted blending	86.30%	72.81%	62.58%	80.41%	75.53%
No CRF	88.78%	96.29%	94.04%	94.92%	93.51%
No verification stopping	99.93%	93.06%	96.60%	**54.89%**	86.12%
